# Acute-on-Chronic Pattern of Isolated Upper Back Pain in a Patient With Acute Coronary Syndrome

**DOI:** 10.7759/cureus.34923

**Published:** 2023-02-13

**Authors:** Yukinori Harada, Taiki Masuyama, Masashi Yokose, Taro Shimizu

**Affiliations:** 1 Department of Diagnostic and Generalist Medicine, Dokkyo Medical University Hospital, Mibu, JPN; 2 Department of Cardiovascular Medicine, Dokkyo Medical University Hospital, Mibu, JPN

**Keywords:** acute coronary syndrome, non-st elevation myocardial infarction, atypical presentation, professional driver, upper back pain

## Abstract

The aim of this case report is to describe the diagnostic pitfalls of acute coronary syndrome in patients with relatively atypical presentation and how we can prevent diagnostic errors in such a patient, particularly focusing on occupational information. A 66-year-old male, a professional taxi driver, presented with severely deteriorated chronic upper back pain on the left side. Furthermore, the upper back pain was exacerbated by changes in position. An orthopedist examined the patient and arrived at a provisional diagnosis of musculoskeletal pain. However, as the patient was concerned about his cardiopulmonary diseases, he visited another physician. Although musculoskeletal pain was still considered as the most possible diagnosis, the physician advised him additional tests for cardiovascular diseases because he had some risk factors such as smoking, hypertension, and dyslipidemia, and the physician thought that “taxi driving” was a high-risk occupation for cardiovascular diseases. Finally, the patient was diagnosed with acute coronary syndrome, and the pain abated soon after percutaneous coronary intervention. Musculoskeletal pain is very common in professional drivers, and isolated upper back pain worsened by changes in position is a characteristic of musculoskeletal disease. However, since professional drivers also have a higher risk of cardiovascular diseases, physicians should consider the coexistence of two types of conditions. This case underscores that if physicians could utilize occupational information to assess patients’ risks, diagnostic accuracy would improve, particularly in patients presenting with atypical symptoms and signs, which are at risk of diagnostic errors.

## Introduction

Generally, in daily clinical practice, the patient’s occupational background does not seem to be regarded as helpful information other than when occupation-related diseases are thought of. However, some occupations such as professional driver can be significant risk factors for some diseases (e.g., cancer, cardiovascular, and pulmonary diseases) [1-3]. Therefore, if physicians could utilize occupational information to assess the patient’s risks, diagnostic accuracy would improve, particularly in patients presenting with atypical symptoms and signs, which are at risk of diagnostic errors. The aim of this case report is to describe the diagnostic pitfalls of acute coronary syndrome in patients with relatively atypical presentation and how we can prevent diagnostic errors in such a patient, particularly focusing on occupational information.

## Case presentation

A 66-year-old male presented at the outpatient department of orthopedics in our hospital with a complaint of upper back pain on the left side. Although the patient noticed the discomfort and back pain while driving a taxi for around a month, he did not visit a doctor because he did not want to take a day off from work. However, the pain suddenly became severe, and the patient developed cold sweat three days before presenting to our hospital. When the pain continued for the next two days, the patient decided to visit the hospital. Other than driving, the pain worsened by motion and while taking a deep inspiration, whereas it lessened by rest. The pain was dull, persistent, and non-radiating. The patient had no symptoms such as chest pain, dyspnea, fatigue, or palpitation. The patient reported that elevated blood pressure, dyslipidemia, and arrhythmia were pointed out on the latest annual health checkup but was not on any medication. His family history revealed that his father suffered from gastric cancer and his mother from colon cancer; there was no family history of cardiovascular diseases. The patient was a current smoker with a history of 48 pack-years and drank 350 mL of beer every day.

The patient was initially examined by an orthopedist. The patient admitted that the intensity of pain decreased on the day of presentation. The pain was experienced in the upper back on the left side. There was no tenderness, knock pain, or rash on the back. The pain increased by lateral flexion of the trunk toward the left but not by any other motions. Plain films of the thoracic and lumbar vertebrae and the ribs were normal. The orthopedist thought that musculoskeletal causes could explain the pain; however, as the patient was also concerned about pulmonary or cardiac diseases, the patient was referred to the department of general medicine on the same day.

On reassessment of the patient at the department, he appeared well. The height and weight were 162 cm and 63 kg, respectively; the body mass index was 24.0. On physical examination, the blood pressure was 136/88 mmHg measured on the right arm, without significant difference between the arms. The pulse rate was 71 beats per minute, respiratory rate was 16 breaths per minute, body temperature was 36.6°C, and oxygen saturation was 98% on ambient air. There were no abnormal lung sounds or murmurs.

Since the patient had pain for approximately one month and had risk factors for lung cancer, such as smoking and taxi driving [1,2], the physician believed that pleural invasion of lung cancer was the most likely cause of the pain. However, considering that the patient also experienced sudden worsening of the pain and the associated cardiovascular risk factors such as smoking, elevated blood pressure, dyslipidemia, and taxi driving [2,3], acute coronary syndrome and aortic dissection were also of concern.

**Figure 1:**
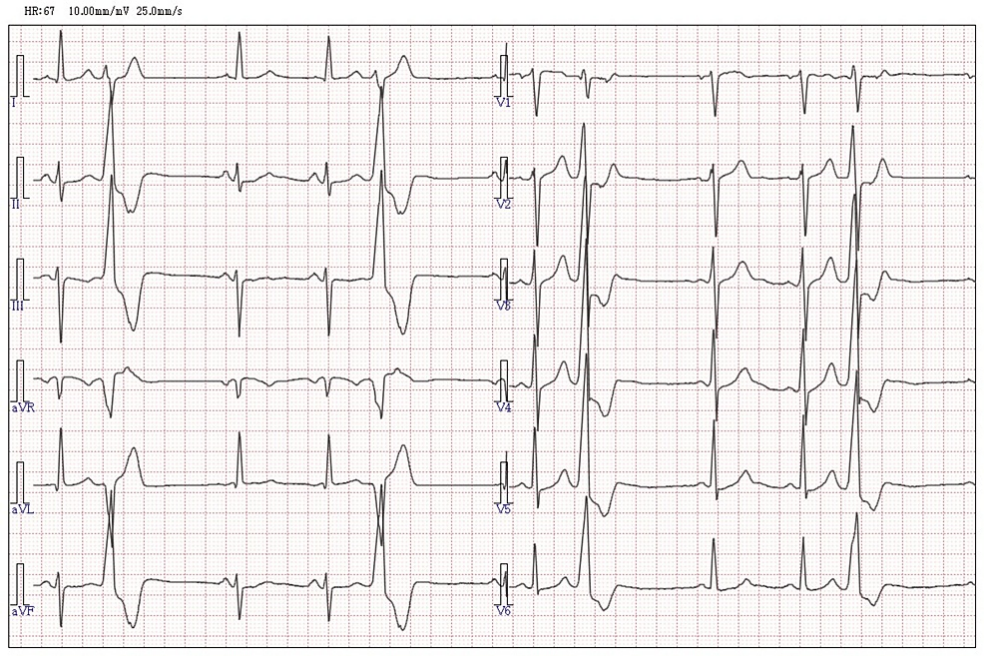
Electrocardiogram on the day of presentation An electrocardiogram showing premature ventricular contractions without ST segment abnormalities

An electrocardiogram showed premature ventricular contractions without ST segment abnormalities (Figure 1).

Chest computed tomography (CT) without contrast showed no evidence of fracture, pleural effusion, pneumothorax, abnormal opacities, or aortic and pulmonary artery dilatation. The probability of being a musculoskeletal pain seemed to increase; however, the high-sensitivity troponin I level turned out to be 150.15 ng/L. Since the patient’s aortic dissection detection risk score was 1 and D-dimer level was below 300 ng/mL, aortic dissection seemed unlikely [4,5]. Other laboratory results included white blood cell count, 7,100/µL; hemoglobin, 15.3 g/dL; platelet count, 26.9/µL; lactate dehydrogenase, 193 U/L; creatine kinase, 79 U/L; total cholesterol, 303 mg/dL; triglyceride, 757 mg/dL; low-density lipoprotein cholesterol, 213 mg/dL; glucose, 102 mg/dL; and glycated hemoglobin, 5.5%. Acute myocardial injury due to coronary artery diseases, myocarditis, or Takotsubo cardiomyopathy were considered as differential diagnoses, and the patient was referred to the department of cardiology for further evaluation.

**Figure 2:**
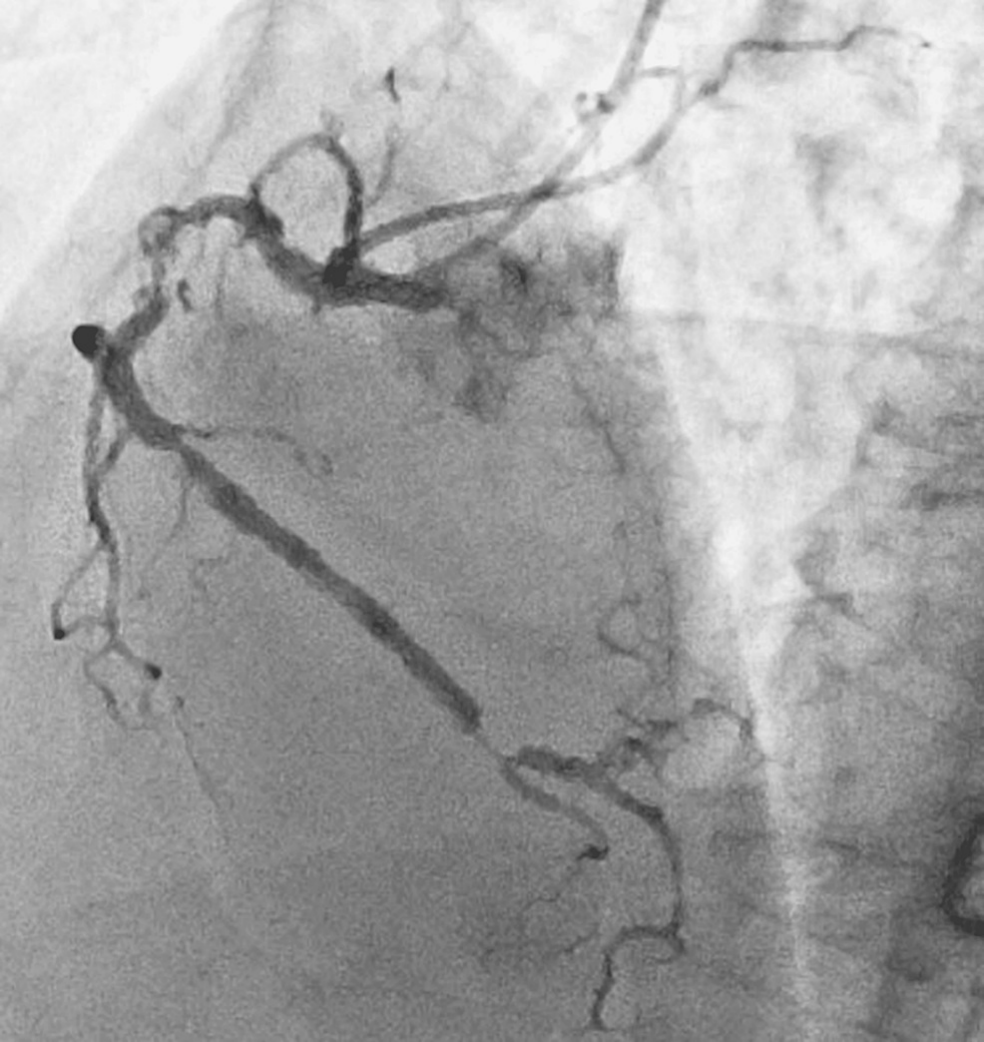
Coronary angiogram Coronary angiogram showing multiple stenoses in the right coronary artery

The patient was evaluated by a cardiologist. Transthoracic echocardiography revealed mild hypokinesis in the anterior-anteroseptal and inferior-anteroseptal regions. Chest and abdominal contrast-enhanced CT ruled out the possibility of aortic dissection and pulmonary embolism, while coronary CT showed stenoses in the right coronary artery. Subsequently, coronary angiography was performed immediately, which showed multiple stenoses in the right coronary artery (Figure 2).

The patient was diagnosed with non-ST elevation myocardial infarction; thus, he underwent percutaneous coronary intervention with drug-eluting stent placement. After the procedure, the pain rapidly disappeared. Although the patient’s condition was well, since the patient needed to complete an in-hospital cardiac rehabilitation program, the patient was hospitalized until day 10.

## Discussion

This was a case successfully managed with a timely correct diagnosis of acute coronary syndrome in a patient with an atypical presentation and with an acute-on-chronic isolated upper back pain. It has been said that only the “typicality” of presentation may lead physicians to make incorrect decisions while discriminating acute coronary syndrome from other diseases [6,7]. Indeed, only three of 13 common symptoms were reported to be predictive of a diagnosis of acute coronary syndrome versus non-acute coronary syndrome [8]. Furthermore, the “typical” presentation of acute coronary syndrome varies with several factors such as age, sex, and comorbidity [6,9]. Therefore, not only symptoms but also the risk factors of patients can impact the decision of correct diagnosis of acute coronary syndrome with an “atypical” presentation.

Regarding the background of patients, occupational information can sometimes be a key factor in the assumption of the risk factors for specific diseases. In this case, the patient’s occupation, “taxi driver,” prompted the physician to consider cancer and cardiovascular diseases, which resulted in the correct diagnosis. Meanwhile, musculoskeletal upper back pain being common among professional drivers [10], the information “taxi driver” would have misled to an incorrect diagnosis if the orthopedist would have anchored the patient to musculoskeletal pain and did not refer him to another physician. This case illustrates the challenging situation of arriving at a correct diagnosis in patients who engage in a specific occupation that carries high risks for several diseases, such as a professional driver.

Upper back pain is a common symptom among professional drivers, including taxi drivers. In a previous systematic review, approximately 25% (up to 60%) of professional drivers experienced musculoskeletal upper back pain [10]. Based on the data, the information “taxi driver” seems to be a driving force for physicians to assume musculoskeletal pain as the most likely cause in patients with upper back pain. On the other hand, upper back pain is also a common type of pain in patients with acute coronary syndrome (around 20%) [8]. Furthermore, taxi drivers have been known to be a high-risk population for cardiovascular diseases including ischemic heart disease [2,3]. From this viewpoint, the information “taxi driver” seems to be a driving force for physicians to suspect cardiovascular diseases in patients with upper back pain.

However, a recent study conducted in Japan suggested that the risk of acute myocardial infarction was not higher in taxi drivers compared to other occupations after adjusting several factors such as smoking history [11]. Indeed, it is also reported that taxi drivers have multiple background risk factors for cardiovascular diseases such as hypertension, diabetes, dyslipidemia, drinking alcohol, smoking, and insufficient physical activity [12], as some of which were observed in this case. These results indicated that the information “taxi driver” itself may not be an independent risk for acute coronary syndrome but rather the trigger information for physicians to take a further history of risk factors for cardiovascular disease, which can result in the right direction for the correct diagnosis of the acute coronary syndrome. Hence, when a piece of clinical information carries multiple risks, it is inevitable for physicians to be aware of every single risk factor and to be careful not to anchor it to just one risk factor. Furthermore, precise, thorough assessment by depicting a whole picture of a patient can be a turning point for whether physicians can utilize occupational information as a driving force for the correct diagnosis of the cause of upper back pain in patients who are taxi drivers.

In this case, the pain of the patient developed around one month prior, and the characteristics seemed to be consistent with musculoskeletal pain; however, the pain suddenly worsened and was accompanied by cold sweat, which indicated that a new additional event occurred in the patient with a chronic condition. Since the pain abated soon after percutaneous coronary intervention, the suddenly worsened upper back pain could have been mainly caused by acute myocardial infarction. On the other hand, since the exacerbation of pain with changes in position in this patient was atypical for acute coronary syndrome [13], the sudden worsening of the upper back pain in this patient was also thought to be partly derived from musculoskeletal causes. Therefore, the acute upper back pain in this patient may have been explained by the coexistence of acute myocardial infarction and musculoskeletal disease.

## Conclusions

Acute coronary syndrome is a common and critical disease that physicians should not miss. However, it is well known that there are wide variations of clinical manifestations of acute coronary syndrome, which can easily lead physicians to misdiagnose. Increasing awareness of important information suggesting acute coronary syndrome, which can reduce the risk of cognitive biases such as anchoring and premature closure, is warranted to avoid diagnostic errors of acute coronary syndrome. In this case, all physicians engaged in the care of a patient could successfully use medical histories such as smoking, hypertension, dyslipidemia, and the “driver” information for a timely correct diagnosis of acute myocardial infarction. In summary, physicians should know that easily missed information, such as occupation, can sometimes drive the correct diagnosis of specific diseases. Such awareness may help physicians correctly diagnose patients with atypical presentations.
